# Ixekizumab for the treatment of psoriatic arthritis: an Italian multicentric retrospective observational study

**DOI:** 10.1038/s41598-026-37835-x

**Published:** 2026-02-19

**Authors:** Stefano Gentileschi, Riccardo Terribili, Carla Gaggiano, Elisa Fiorentini, Laura Cometi, Cosimo Cigolini, Fabio Massimo Perrotta, Silvia Scriffignano, Luca Di Cato, Anna Panaccione, Laura Niccoli, Fabrizio Cantini, Maurizio Benucci, Francesca Li Gobbi, Andrea Delle Sedie, Antonio Vitale, Ennio Lubrano, Bruno Frediani, Serena Guiducci

**Affiliations:** 1 Rheumatology Unit, Department of Medicine, Surgery and Neurosciences, University of Siena, Siena University Hospital, viale Mario Bracci 16, 53100 Siena, Italy; 2 Department of Experimental and Clinical Medicine, Division of Rheumatology, University of Florence, 50134 Florence, Italy; 3 Rheumatology Unit, Department of Clinical and Experimental Medicine, University of Pisa, 56124 Pisa, Italy; 4 Academic Rheumatology Unit, Department of Medicine and Health Sciences “Vincenzo Tiberio”, University of Molise, 86100 Campobasso, Italy; 5 Rheumatology Unit, Santa Maria General Hospital, 05100 Terni, Italy; 6 Rheumatology Unit, S. Stefano Hospital, 59100 Prato, Italy; 7 Rheumatology Unit, S. Giovanni Di Dio Firenze Hospital, 50134 Florence, Italy

**Keywords:** Psoriatic arthritis, Ixekizumab, Interleukin-17, Real-world evidence, Inflammation, Autoimmunity, Rheumatology, Rheumatic diseases, Outcomes research

## Abstract

Ixekizumab (IXE), an IL-17 A inhibitor, demonstrated efficacy in clinical trials in patients with psoriatic arthritis (PsA), and favorable data have emerged from real-world evidence studies on psoriasis as well. However, real-world data specific to PsA remain limited. This study aims to assess IXE effectiveness in reducing disease activity in patients with PsA and determine its drug retention rate (DRR) over 24 months. The secondary aim is to identify factors potentially affecting long-term persistence on therapy. A retrospective observational study was conducted. Consecutive adult patients meeting the CASPAR criteria for PsA and treated with IXE for ≥ 3 months were included. Patients were evaluated at regular intervals on a routine clinical basis for disease activity and quality of life assessment. 132 patients (78 females, 54 males; mean age 59.1 ± 11.9 years) were included. At baseline, the median (IQR) Disease Activity in Psoriatic Arthritis (DAPSA) was 16.2 (7.7), and the visual analogue scale (VAS) for pain was 6.5 (3.0). In patients with axial involvement, the median (IQR) Ankylosing Spondylitis Disease Activity Score – C-reactive protein (ASDAS-CRP) was 3.4 (1.3), and the Bath Ankylosing Spondylitis Disease Activity Index (BASDAI) was 5.0 (1.5). IXE treatment was associated with statistically significant reductions in DAPSA (*p* < 0.001), ASDAS-CRP (*p* < 0.001), BASDAI (*p* < 0.001), VAS-pain (*p* < 0.001), Health Assessment Questionnaire (HAQ) (*p* < 0.001), erythrocyte sedimentation rate (*p* = 0.014), and CRP (*p* = 0.004) over 24 months. At 24 months, remission and low disease activity, according to DAPSA thresholds, were achieved by 46.7% and 93.3% of patients, respectively, while Very Low Disease Activity and Minimal Disease Activity criteria were met by 16.0% and 34.0%, respectively. IXE DRR was 82.1%, 76.3%, and 73.3% at 12, 18, and 24 months, respectively, with lower values in female patients (*p* = 0.036). No differences were observed in IXE DRR when stratifying the cohort by axial involvement (*p* = 0.84), prior exposure to biologics or tsDMARDs (*p* = 0.68), or body mass index (BMI) categories (*p* = 0.48). In conclusion, IXE enabled rapid and sustained disease control in patients with PsA across multiple disease domains. The high DRR supports its long-term use, regardless of prior biologic exposure or BMI. Gender differences in IXE treatment response may warrant further exploration.

## Introduction

Psoriatic arthritis (PsA) is a chronic inflammatory disease with multisystem involvement, primarily affecting skin and nails, peripheral and axial joints, and periarticular tissues such as entheses, tendons, capsules, and ligaments^1^. Its presentation is highly heterogeneous and often accompanied by comorbidities, including metabolic syndrome, cardiovascular disease, and depression, as well as additional organ involvement, such as Crohn’s disease (CD) and uveitis^2^. Indeed, the complex manifestations of PsA, coupled with its systemic nature, have led to the recent adoption of the term “psoriatic disease”, which more accurately reflects the shared pathogenetic mechanisms driving its varied clinical presentations^3^.

A critical feature of PsA is the relationship between psoriasis (PsO) and musculoskeletal inflammatory symptoms, with up to 30% of PsO patients potentially developing arthritis, which may appear before cutaneous symptoms or concomitantly^4^. Although the exact pathogenetic mechanisms underlying PsA are not fully understood, environmental factors are likely to interact with genetic predisposition leading to abnormal activation of both the innate and adaptive immune systems. Central to this immune dysregulation is interleukin (IL)-17, whose production is primarily driven by the activation of Th17 lymphocytes through mediators such as transforming growth factor beta (TGF-β), IL-6, IL-23. The release of IL-17 significantly contributes to the amplification of the inflammatory response, with synovial fibroblasts, chondrocytes and osteoclasts activation and the stimulation of the nuclear factor kappa-light-chain-enhancer of activated B cells (NF-kB) pathway further exacerbating the process^5^.

In this context, the introduction of the IL-17 blocking agents alongside conventional disease-modifying anti-rheumatic drugs (cDMARDs) and anti-tumor necrosis factor (TNF) treatments, marked a significant advancement in the management of both PsO and PsA. Notably, Ixekizumab (IXE), a humanized monoclonal IgG4 antibody targeting the IL-17 A isoform, was licensed by the European and American regulatory agencies for the treatment of these conditions in 2016 and 2017 respectively.

Clinical trials have consistently demonstrated IXE efficacy in controlling disease activity across multiple PsA domains, compared to placebo in treatment-naive and drug-experienced patients, while maintaining a robust safety profile (SPIRIT-P1, SPIRIT-P2, SPIRIT-P3, and SPIRIT-H2H)^6–11^.

These encouraging results have been replicated and confirmed in real-life studies, which also reported high drug retention rates (DRR)^12–14^. However, unlike the registration trials, most real-life data focus on the use of IXE in patients with PsO, with specific evidence for PsA still being limited^8^.

Therefore, we have designed a retrospective study to assess the effectiveness of IXE in controlling disease activity in patients with PsA, and to evaluate the long-term persistence in therapy in a multicenter cohort.

## Methods

### Aim of the study and patient selection

A retrospective multicentric observational study was conducted at seven Italian rheumatology centers.

The primary aims of the study were (1) to assess IXE effectiveness in reducing disease activity in patients with PsA, and (2) to determine IXE DRR over 24 months. The secondary aim of the study was to identify any factors that might influence the DRR of IXE in patients with PsA.

All consecutive adult patients with PsA treated with IXE in routine clinical practice from 2018 to 2024 at the participating rheumatology centers were identified through review of electronic medical records and outpatient clinic databases. Patients were eligible for inclusion if they: (1) were ≥ 18 years of age; (2) fulfilled the 2006 Classification Criteria for Psoriatic Arthritis (CASPAR)^15^; and (3) received IXE for the treatment of PsA for a minimum of 3 months^8^. Patients were excluded if no baseline clinical assessment was available or IXE was prescribed for indications other than PsA.

Given the retrospective nature of the study, a formal a priori sample size calculation was not performed. The sample size was determined by the number of eligible patients treated with IXE during the study period across participating centers. The resulting cohort size was considered adequate for analyzing longitudinal disease activity, treatment persistence, and exploratory subgroup analyses, and was comparable to or larger than those of previously published real-world studies in PsA^12–14^.

### Disease activity assessment

Disease activity was measured using Disease Activity in Psoriatic Arthritis (DAPSA). Ankylosing Spondylitis Disease Activity Score (ASDAS)- C-reactive protein (CRP) and Bath Ankylosing Spondylitis Disease Activity Index (BASDAI) were used to evaluate disease activity in patients with axial involvement. The visual analogue scale (VAS) for pain was also assessed^8,13,14^. To obtain a more comprehensive picture of disease control across the various domains affected by PsA manifestations, the criteria for Minimal Disease Activity (MDA) were also evaluated. To this aim, enthesitis was clinically evaluated as present or absent. The Health Assessment Questionnaire (HAQ) was used to assess overall quality of life, indirectly measuring the impact of the disease on the ability to carry out daily activities^16^.

DAPSA is a clinimetric index used to assess disease activity in PsA^17^. It includes the Swollen Joint Count (SJC) and Tender Joint Count (TJC) measured on 68/66 joints, the patient’s VAS pain score, the Patient Global Assessment (PtGA), and CRP levels. DAPSA classifications validated for PsA include: ≤4 (remission, REM), > 4–≤14 (low disease activity, LDA), > 14–≤28 (moderate disease activity) and > 28 (high disease activity).

Axial involvement was defined based on the treating rheumatologist’s clinical assessment, supported by imaging findings when available. Clinical evaluation was consistent with inflammatory back pain features as defined by the ASAS expert framework^24^, while imaging evidence of axial spondyloarthritis (axSpA) (radiographs and/or MRI) was recorded when present. ASDAS and the BASDAI are two key indices used to assess disease activity in axSpA^18^. ASDAS is derived from five disease variables measured on 10 cm VAS scales, along with CRP levels, while BASDAI consists of six disease variables, also measured on 10 cm VAS scales. Given the lack of validated axial PsA activity instruments, ASDAS-CRP and BASDAI were used pragmatically to describe axial symptom burden, acknowledging their limitations in this context.

Very Low Disease Activity (VLDA) is defined when all 7 of the following targets are achieved: TJC ≤ 1, SJC ≤ 1, enthesitis count ≤ 1, skin involvement (Psoriasis Area and Severity Index (PASI) ≤ 1 or body surface area (BSA) ≤ 3%), function (measured by the HAQ) ≤ 0.5, patient’s global VAS on a 100 mm scale ≤ 20, and patient pain VAS on a 100 mm scale ≤ 15. If 5 out of 7 criteria are met, the patient is classified as being in MDA^19^.

When collecting information about reasons of IXE discontinuation, the definitions of primary or secondary inefficacy were applied based on musculoskeletal outcomes only, while inefficacy on the cutaneous domain was eventually collected separately as “other reasons for discontinuation”.

### Therapeutic scheme and follow-up

IXE was administered to all patients, according to the Group for Research and Assessment of Psoriasis and Psoriatic Arthritis (GRAPPA) and the European Alliance of Associations for Rheumatology (EULAR) recommendations^20,21^. The licensed therapeutic scheme for PsA was employed, which is IXE 160 mg subcutaneously at the start, followed by 80 mg at 4-week intervals.

Demographics, clinical, and laboratory data from PsA patients were recorded at baseline (T0, start of IXE treatment), followed by assessments at 3 and 6 months, and every 6 months thereafter, extending through a 24-month observational period on a routine clinical basis. When assessments were not conducted exactly at the predefined timepoints, data were assigned to the nearest timepoint provided that an examination was performed within ± 15 days.

### Statistical analysis

Statistical analysis was performed by using JASP open-source statistics package version 0.18.3. Descriptive statistics included sample sizes, mean and standard deviation (SD) or median and interquartile range (IQR). Shapiro-Wilk test was used to assess normality distribution of data. Differences in paired continuous variables were analyzed by repeated measured ANOVA (non-parametric Friedman’s test) and Conover’s post-hoc test. For longitudinal outcomes, the number of participants with available data at each time point was reported, and primary analyses were conducted in participants with complete longitudinal measurements to ensure transparency and a conservative approach. To evaluate the robustness of the results and mitigate potential bias due to incomplete follow-up, linear mixed models were subsequently performed on the full cohort as sensitivity analyses. Differences in paired nominal dichotomic variables were assessed by McNemar’s test, including only participants with available data at the two relevant time points for each comparison. Nevertheless, proportions of patients achieving the outcomes were also reported based on the total cohort for transparency. Time-to-event analysis with DRR calculation was performed by Kaplan-Meier method, with the event being drug discontinuation for any reason except for disease remission. The survival curves were compared according to specific factors by the Log-Rank test. The threshold for statistical significance was set to *p* < 0.05 and all p-values were two-sided.

### Ethics

All patients signed a “Patient Consent Form” in full knowledge of how their data would be used.

The study was conducted in accordance with the declaration of Helsinki and was approved by the Ethics Committee of the University of Siena (protocol RHELABUS 22271).

## Results

### Baseline characteristics of the cohort

A total of 132 patients (78 females/54 males) with a mean age ± SD of 59.1 ± 11.9 years [23.3–83.1] were included. Based on body mass index (BMI) values, 60/118 patients (50.9%) were classified as overweight and 29/118 (24.6%) as obese. While 128 patients (97.0%) presented peripheral joint involvement, 48 patients (36.4%) had also axial involvement. PsO was present in 104 patients (78.8%); among patients without current skin PsO, PsA classification was supported by other CASPAR features, including a documented family history of PsO where available. Nail PsO was observed in 8 of 69 patients (11.6%) with available data. History of dactylitis was reported in 27 patients (20.5%), and enthesitis in 59 (44.7%). Lastly, 2 patients (1.5%) had associated ocular involvement, and the same proportion had intestinal involvement. Seventy-six patients (57.6%) had previously been treated with at least one biologic/targeted synthetic DMARDs (b/tsDMARDs), of whom 25 (18.9%) experienced a primary inefficacy of anti-TNF-α treatment. At baseline, the median (IQR) disease activity was 16.2 (7.7) [0–40.0] according to the DAPSA index (moderate activity), and the median (IQR) VAS-pain score was 6.5 (3.0) [1.0–10.0]. In patients with axial involvement, the median (IQR) values of ASDAS-CRP and BASDAI were 3.4 (1.3) [0–5.1] and 5.0 (1.5) [0–9.3], indicating high and active disease, respectively. Baseline characteristics of the cohort are detailed in Table 1.

**Table 1:** Clinical presentation and demographic features.

Demographic features	Distribution
Age (years), mean ± SD [min–max]	59.1 ± 11.9 [23.3–83.1]
Age at onset (years), mean ± SD [min–max]	48.0 ± 12.1 [16.0–79.0]
Age at diagnosis (years), mean ± SD [min–max]	50.1 ± 11.5 [17.0–79.0]
BMI, mean ± SD [min–max] (n. available observations)	27.1 ± 5.0 [18.8–50.8] (*n* = 118)
Male sex, n. (%)	54 (40.9)
Duration of IXE treatment (months), median (IQR) [min–max]	18.0 (18.0) [3.0–24.0]
**Clinical features**	**N. patients (%)**
Axial involvement	48 (36.4)
Peripheral involvement	128 (97.0)
Dactylitis	27 (20.5)
Enthesitis	59 (44.7)
Psoriasis	104 (78.8)
Nail psoriasis (n. available observations)	8 (11.6) (*n* = 69)
Gut Involvement	2 (1.5)
Ocular Involvement	2 (1.5)
Fibromyalgia (n. available observations)	23 (17.6) (*n* = 131)
Hypercholesterolemia	51 (38.6)
Arterial Hypertension	35 (26.5)
Hyperuricemia	14 (10.6)
**Disease activity**	**Median (IQR) [min**–**max]**
DAPSA	16.2 (7.7) [0–40.0]
ASDAS-CRP	3.4 (1.3) [0–5.1]
BASDAI	5.0 (1.5) [0–9.3]
VAS pain	6.5 (3.0) [1.0–10.0]
HAQ	0.96 (0.8) [0–3.0]
**Previous treatment**	**N. patients (%)**
Prior b/tsDMARDs use	76 (57.6)
TNFi exposure	74 (56.1)
TNFi primary non-responders	15 (11.4)
TNFi secondary non-responders	8 (6.1)

Percentages are calculated on the entire cohort (*n* = 132) unless otherwise specified. For variables with missing data, the number of available observations is reported. BMI was calculated from baseline weight and height; implausible height values were treated as missing for BMI-derived analyses.

*ASDAS-CRP* Ankylosing spondylitis disease activity score-C reactive protein, *BASDAI* bath Ankylosing spondylitis disease activity index, *b/tsDMARD* biologic/targeted synthetic disease modifying antirheumatic drugs, *BMI* body mass index, *DAPSA* disease activity in psoriatic arthritis, *HAQ* health assessment questionnaire, *IXE* ixekizumab, *IQR* interquartile range, *SD* standard deviation, *TNFi* tumor necrosis factor inhibitors, *VAS* visual analogue scale.

### Effectiveness of IXE in controlling disease activity

IXE was administered as monotherapy in 101 cases (76.5%) or in combination with a cDMARD in 29 cases (21.9%). The median treatment duration (IQR) was 18.0 (18.0) months [3.0–24.0].

**Fig. 1:**
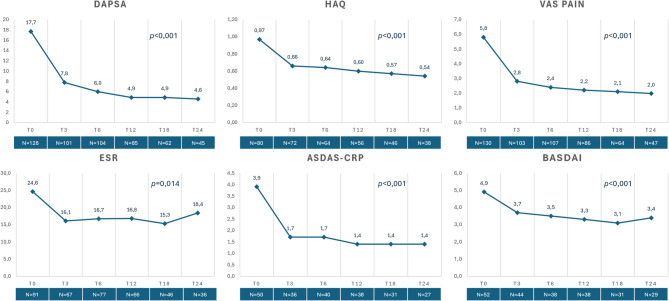
Variation of disease activity indexes over 24 months of follow-up with IXE treatment. The number of patients with available information at each time point is reported. List of abbreviations: *ASDAS-CRP* Ankylosing spondylitis disease activity score-C-reactive protein, *BASDAI* bath Ankylosing spondylitis disease activity index, *DAPSA* disease activity in psoriatic arthritis.

At the repeated measured ANOVA analysis, a statistically significant improvement was observed over 24 months of treatment in the median levels of ESR (*p* = 0.014), CRP (*p* = 0.004), VAS pain (*p* < 0.001), HAQ (*p* < 0.001), DAPSA (*p* < 0.001), ASDAS-CRP (*p* < 0.001) and BASDAI (*p* < 0.001) (Fig. 1). Linear mixed models were applied to all longitudinal outcomes based on the entire cohort as a sensitivity analysis, confirming statistically significant improvements over 24 months of treatment in ESR (*p* < 0.001), CRP (*p* < 0.001), VAS pain (*p* < 0.001), HAQ (*p* < 0.001), DAPSA (*p* < 0.001), ASDAS-CRP (*p* < 0.001) and BASDAI (*p* < 0.001).

At the last follow-up available (T24), the median (IQR) DAPSA score in the whole cohort was 4.3 (3.7) [0.2–18.0]; in the subgroup of patients with axial involvement, the median (IQR) ASDAS-CRP score was 1.3 (0.2) [0.6–3.3] and the median (IQR) BASDAI score was 3.0 (1.2) [0–6.2]. In detail, a statistically significant improvement was observed at the Conover’s Post Hoc Comparisons between T0 and T3 in the case of DAPSA (*p* < 0.001), ASDAS-CRP (*p* = 0.005), BASDAI (*p* = 0.013), HAQ (*p* < 0.001), VAS pain (*p* < 0.001), CRP (*p* = 0.008), and ESR (*p* = 0.02), but not between T3 and T6 nor between T6 and T12 (*p* > 0.05 for all comparisons).

The improvement of DAPSA score during the treatment period was not influenced by the presence of psoriasis (*p* = 0.188 within groups comparison).

**Fig. 2:**
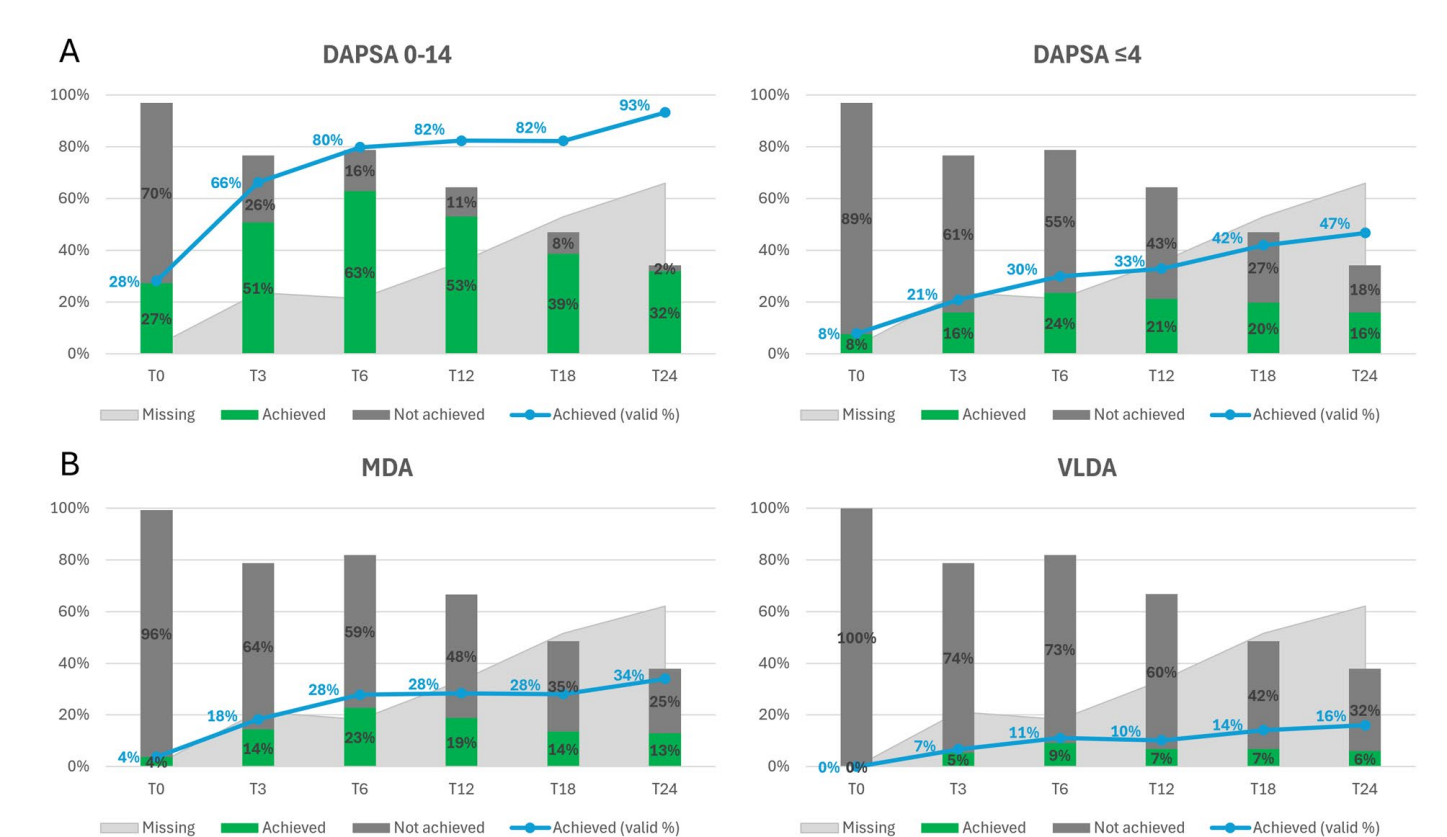
Frequency of DAPSA low disease activity, DAPSA remission (**A**), minimal disease activity or very low disease activity (**B**) achievement over 24 months of follow-up with IXE treatment. The frequency of outcome achievement based on available information (valid %) is reported in blue. Other frequencies reported as bars are based on the entire cohort (actual %). *DAPSA* disease activity in psoriatic arthritis, *MDA* minimal disease activity, *VLDA* very low disease activity.

Remission and Low Disease Activity (LDA) according to the DAPSA were achieved by 20.8% and 66.3% of patients at T3 (*p* = 0.06 and *p* < 0.001, respectively), 29.8% and 79.8% at T6 (*p* < 0.001 for both), 32.9% and 82.3% at T12 (*p* = 0.003 and *p* < 0.001, respectively), and 46.7% and 93.3% at T24 (*p* = 0.07 and *p* = 0.53, respectively) (Fig. 2A).

VLDA and MDA were achieved by 6.7% and 18.3% of patients at T3 (*p* = 0.02 and *p* < 0.001, respectively), 11.1% and 27.9% at T6 (*p* < 0.001 for both), 10.2% and 28.4% at T12 (*p* = 0.003 and *p* < 0.001, respectively), and 16.0% and 34.0% at T24 (*p* = 0.008 and *p* < 0.001, respectively) These percentages refer to the entire cohort and not only to the patients in treatment at the respective timepoint (Fig. 2B).

### Long-term persistence on IXE treatment

**Fig. 3:**
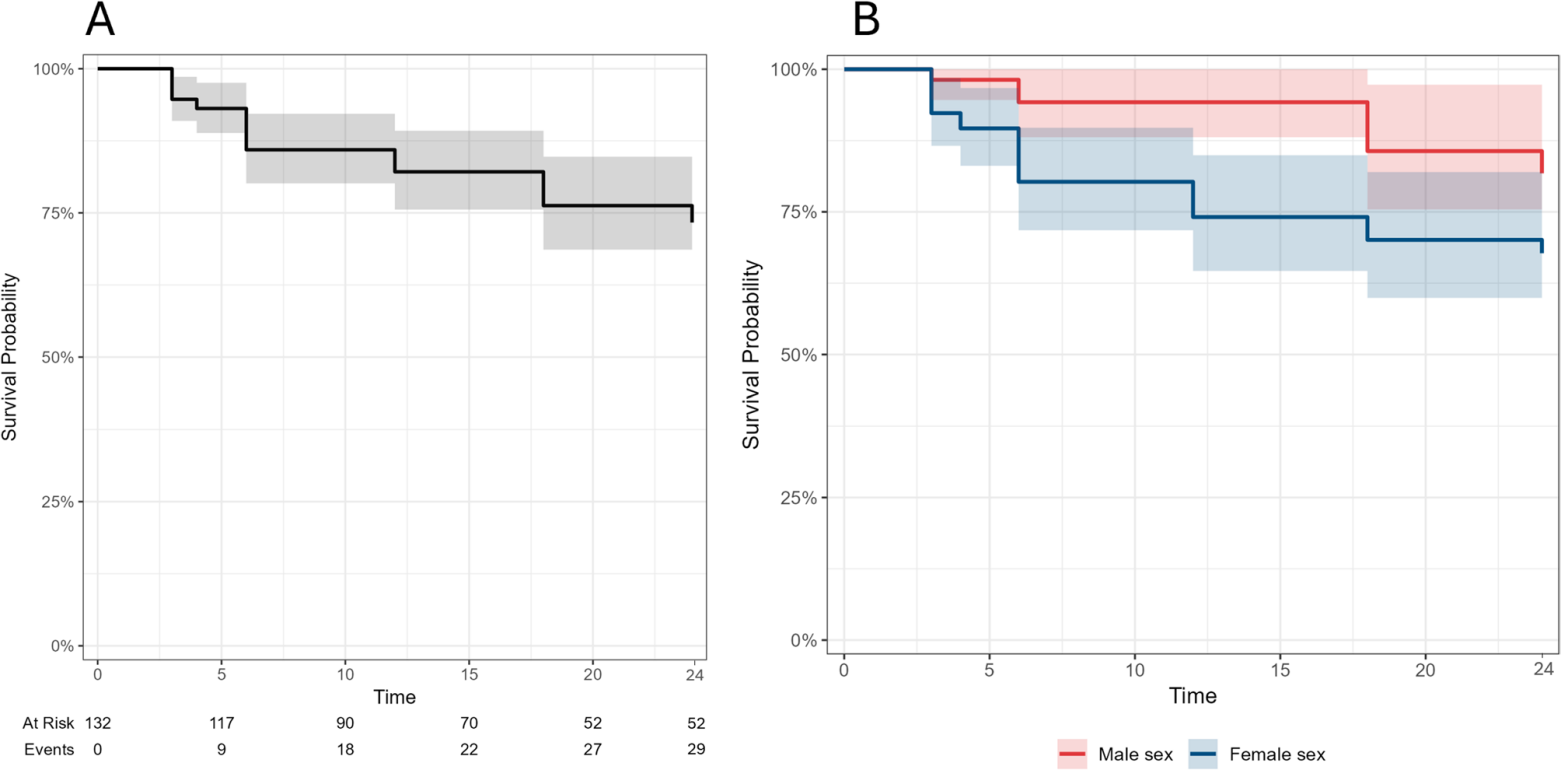
Survival curve of IXE over 24 months of follow-up (**A**). When stratifying the cohort according to sex, the drug retention rate of IXE was higher in male than female patients (*p* = 0.036 at the Log-rank test) (**B**).

The DRR of IXE at 12, 18, and 24 months were 82.1%, 76.3%, and 73.3%, respectively (Fig. 3A). When stratified by sex, women showed a lower DRR compared to men (*p* = 0.036) (Fig. 3B).

No differences were observed when stratifying the cohort by the presence of axial involvement (*p* = 0.84), prior exposure to b/tsDMARDs (*p* = 0.68) and TNF inhibitors specifically (*p* = 0.57), presence of psoriasis (*p* = 0.05), or different BMI categories (*p* = 0.48).

At the end of the observation period, 29 patients (22%) had discontinued treatment with IXE. Specifically, 9 cases (6.8%) of adverse events (AEs) were reported (injection site skin reaction in 5 cases (3.8%) and mild infections in 4 cases (3%)), while 23 patients (17.4%) discontinued treatment due to primary (i.e. lack of efficacy) or secondary inefficacy (i.e. loss of efficacy), respectively in 15 cases (11.4%) and 8 cases (6%) (an adverse event was reported along with inefficacy in 3 patients). None discontinued IXE due to inefficacy on psoriasis. Of note, no exacerbation of gastrointestinal symptoms was observed in the 2 patients with an associated diagnosis of CD.

## Discussion

The results from the present study support IXE effectiveness in the management of PsA in a real-world setting, with rapid and sustained improvements across key disease domains. Highly encouraging findings were also observed for DRR over a 24-month period of treatment, regardless of patients clinical and treatment variables.

The clinical characteristics of PsA in our cohort largely align with the existing literature, especially considering the reported wide variability in the manifestations of dactylitis, enthesitis, and axial involvement^22–25^. The high prevalence of overweight and obesity, as highlighted by BMI values, was expected^26^. Obesity is one of the most common comorbidities in PsA, supporting the theory that adipose tissue plays a role not only in metabolic processes but also in inflammatory and autoimmune mechanisms.

Concerning clinical effectiveness, IXE treatment allowed a steady disease control across all disease domains in our patients, with a rapid onset of response, observed at 3 months and maintained thereafter. In fact, we registered a considerable reduction in key clinimetric indexes, including DAPSA, ASDAS-CRP and BASDAI. These improvements were most notable in both peripheral and axial joint symptoms. Additionally, a similar trend was observed in the MDA composite score, reflecting significant progress not only in joint involvement but also in skin symptoms and enthesitis (VLDA reached by 16% of patients at T24 and MDA by 34% of patients).

Such data are especially important since, despite the inclusion of PsA patients in various real-world cohort studies involving IXE, specific PsA-related efficacy outcomes (beyond the PASI score used for the assessment of PsO) have been analyzed to a limited extent^14,27–33^. In particular, it is worth mentioning the results from a 2024 multicentric prospective registry analysis from the Gruppo Italiano Studio Early Arthritis (GISEA) collecting 223 PsA patients. The authors reported a significant improvement within 6 months of IXE treatment for TJC and SJC and DAPSA score, persisting up to 24 months^14^.

Other recent real-world reports have further contributed to the evidence on IXE, although with different target populations and study objectives. Building on this context, our findings extend the existing real-world literature on IXE in two main ways. Chiricozzi et al. (LOTIXE) evaluated long-term IXE effectiveness in moderate-to-severe plaque PsO, primarily using dermatologic endpoints (e.g., PASI and DLQI) in a responder-enriched population requiring ≥ 12 months of continuous treatment^30^, whereas our study focuses on a CASPAR-classified PsA cohort and evaluates longitudinal musculoskeletal disease activity with validated disease activity indexes (e.g., DAPSA) together with PROs and functional measures. Furthermore, Bellis et al. investigated IXE drug survival and predictors of discontinuation in a monocentric PsA cohort focusing on DRR^12^, while our multicenter dataset integrates both multi-domain effectiveness trajectories and 24-month treatment persistence, including patients who discontinued during follow-up, thereby reflecting routine rheumatology practice closely.

Furthermore, a notable finding in our group was the favorable impact of IXE on QoL, as measured by the HAQ score, which is of particular importance given the well-documented disease burden associated with PsA^7,34,35^. IXE also significantly reduced pain, as demonstrated by changes in the VAS score. This is a critical finding, given that pain is a predominant symptom in PsA, and its control is essential for effective disease management. There is growing evidence of central sensitization mechanisms leading to chronic pain in patients with PsO and PsA^36^. Indeed, we recorded a substantial prevalence of 17.6% of fibromyalgia in our cohort. Considering the involvement of IL-17 in neuroinflammation and neuropathic pain, anti-IL-17 therapies, including IXE, have been hypothesized to play a role in modulating chronic pain, supported by encouraging preclinical results^37^. However, these mechanistic considerations are speculative and cannot be inferred from the present observational data.

More than half of the patients in our cohort had already been treated with b/tsDMARDs, a substantial proportion of whom had used anti-TNF. Indeed, a detailed subgroup analysis of both patient groups showed no significant differences in DRR achievement based on the type of previously administered biologic drug, nor was there an overall reduction in DRR compared to biologic-naïve patients. This observation is consistent with other available studies, although real-world data suggest that biologic-naïve patients may experience slightly greater efficacy, albeit with minimal differences from pre-treated patients^7,31^. The overall evidence thus offers reassuring support for the effective and lasting use of IXE even in later lines of therapy in PsA.

A considerable portion of our cohort had axial involvement. In those patients ASDAS-CRP and BASDAI scores decreased over follow-up, which was associated with improvement in axial disease activity measures in this observational setting. This finding is particularly notable given the limited data on IL-17 inhibitors in psoriatic spondylarthritis^38^. It is becoming increasingly evident that radiographic(r)-axSpA and psoriatic axSpA represent distinct pathophysiological entities^24,39^. This distinction highlights the need for dedicated research into psoriatic axSpA, rather than relying on data extrapolated from studies on non r- and r-axSpA. Furthermore, ASDAS-CRP and BASDAI are validated for axSpA rather than axial PsA, and their use in axial PsA should be interpreted cautiously.

Most patients remained on IXE throughout the observation period, with a high persistence rate of 73.3% at the 24-month follow-up. Treatment discontinuation was primarily due to primary or secondary inefficacy, followed by mild AEs such as injection site reactions or non-severe infections. Overall, IXE was shown to be safe and well-suited for long-term treatment. Real-world data from insurance claims databases and cohort/registry studies on patients with PsO, PsA, and axSpA reported IXE treatment persistence rates exceeding 70%, with follow-up periods extending up to 24 months^40–43^, with overall superiority in retention rates compared to other biologics (except ustekinumab)^44–46^. Two recent studies evaluating IXE use in PsA patients reported DRR of 43.8% at 38 months and 57% at two years, with similar proportions of discontinuation due to inefficacy or mild AEs^12,13^. Braña et al. identified depression and prior methotrexate use as predictors of treatment discontinuation^13^.

In our cohort, women had a significantly lower DRR compared to men, although no differences were observed based on axial involvement, prior biologic exposure, or BMI class. This finding is consistent with other studies, which have shown that women with PsA generally report worse patient-reported outcomes (PROs) related to quality of life, fatigue, and pain, and have less favorable responses to biologic treatment compared to men^47^. Interestingly, although a recent pooled data analysis on the phase 3 SPIRIT-P1 and SPIRIT-P2 RCTs highlighted higher DAPSA improvements in males^48^, attributable to a greater therapeutic effect in this sex, Chimenti et al. found IXE to be equally effective in both sexes after 24 months^14,48^. This discrepancy could be possibly attributed to the increased pain sensitivity and risk for clinical pain observed among women compared to men^49^, as well as the higher prevalence of fibromyalgia in the female population^50^, which may contribute to a greater persistence of symptoms in women, even after treatment and despite the achievement of remission or improvement in disease manifestations. Studies specifically designed on PsA patients with concomitant fibromyalgia could help clarify these intriguing aspects.

Furthermore, the lack of correlation between BMI values and the efficacy of IXE treatment is not unexpected, as previous studies have reported similar findings consistent with other anti-IL-17 therapies^51,52^. Conversely with other classes of biologic drugs, such as anti-TNF, where body weight has been shown to significantly impact treatment outcomes^52,53^. This observation holds great significance in highlighting the remarkable versatility of IXE treatment, especially in terms of providing a personalized therapy for PsA patients, tailored not only to their specific clinical presentation but also to their unique comorbidity profile.

Regarding the potential impact of psoriasis on IXE effectiveness and long-term drug survival, no clinically meaningful differences were observed in our cohort. Disease activity, as assessed by DAPSA, improved consistently in patients both with and without psoriasis at baseline. Moreover, a significant impact of skin involvement on DRR was not demonstrated; this analysis was limited by marked group imbalance and a low number of events, which reduced statistical power and resulted in unstable survival estimates.

The proportion of patients who discontinued treatment due to AEs was consistent with findings in the literature, with no severe AEs reported^7^. Indeed, injection site reactions and non-severe infections, especially localized candidiasis, have been previously described in clinical trials and real-world studies^11,54^.

Although our data on gastrointestinal involvement is limited due to missing information, in the two patients with CD, IXE did not lead to disease exacerbation, despite previous reports suggesting a potential risk for disease activation, likely related to the crucial role of IL-17 signaling in maintaining intestinal epithelial barrier integrity^55–57^.

The relevance of the findings from our study is supported by its multicentric design and quite large sample size, making it one of the most comprehensive real-world studies on IXE in PsA to date. Furthermore, the detailed evaluation of treatment efficacy across multiple disease domains, including the MDA criteria, provides further valuable insights. The extended follow-up period also strengthens our data, offering a clearer picture of IXE long-term performance. Lastly, the inclusion of a substantial number of patients with psoriatic axSpA, a clinically distinct entity from r-axSpA, adds to the novelty of our findings, contributing to the limited data available on IL-17 inhibitors in this context. Nevertheless, these results should be interpreted with caution, as disease activity was assessed using ASDAS-CRP and BASDAI, indices originally developed and validated for axSpA rather than specifically for psoriatic axSpA.

Our study is limited by its observational and retrospective design, which offers less robust evidence compared to interventional studies and limits causal inference, particularly in the absence of a comparator group. The retrospective design may introduce selection bias potentially influencing both treatment response and retention. Additionally, there was missing data for some variables (e.g., nail psoriasis), and outcome availability decreased over time, which may bias longitudinal estimates. Despite this limitation, the consistency of improvement across multiple disease activity indices, patient-reported outcomes, and inflammatory markers, along with sensitivity analyses results, support the robustness of the observed treatment effect.

## Conclusions

This multicenter study supports the potential efficacy of IXE in managing PsA, pointing to a significant and rapid improvements across joint, skin, and enthesitis domains by the third month of treatment, alongside sustained pain relief and enhanced quality of life. The high DRR at 24 months suggests durable effectiveness across patient profiles, including those previously treated with biologics, regardless of BMI. Although female patients showed lower DRR, consistent with prior studies on gender disparities, the safety profile remained favorable with only mild AEs observed. These findings are consistent with a beneficial role of IXE in the management of PsA and psoriatic spondylitis, while further research on gender effects and comparison with other biologic treatments remains warranted. Given the retrospective observational design and the absence of a comparator group, these findings should be interpreted as associations.

## Data Availability

All data generated or analysed during this study are included in this published article.

## References

[1] Tiwari, V. & Brent, L. H. Psoriatic Arthritis. StatPearls Publishing (2024). 31613490

[2] Novelli, L. et al. Extra-Articular manifestations and comorbidities in psoriatic disease: A journey into the Immunologic crosstalk. *Front. Med. (Lausanne)*. **8**, 737079 (2021). 34631754 10.3389/fmed.2021.737079 PMC8495009

[3] Lubrano, E., Scriffignano, S. & Perrotta, F. M. Psoriatic Arthritis, psoriatic Disease, or psoriatic syndrome? *J. Rheumatol.* **46**, 1428–1430 (2019). 31676545 10.3899/jrheum.190054

[4] Scotti, L., Franchi, M., Marchesoni, A. & Corrao, G. Prevalence and incidence of psoriatic arthritis: A systematic review and meta-analysis. *Semin Arthritis Rheum.* **48**, 28–34 (2018). 29398124 10.1016/j.semarthrit.2018.01.003

[5] Azuaga, A. B., Ramírez, J. & Cañete, J. D. Psoriatic arthritis: pathogenesis and targeted therapies. *Int. J. Mol. Sci.* 24. (2023). 10.3390/ijms24054901 PMC10003101 36902329

[6] Deodhar, A. A. et al. Safety of Ixekizumab in patients with psoriatic arthritis: data from four clinical trials with over 2000 patient-years of exposure. *Ann. Rheum. Dis.* **81**, 944 (2022). 35393269 10.1136/annrheumdis-2021-222027 PMC9209663

[7] Coates, L. C. et al. Withdrawing Ixekizumab in patients with psoriatic arthritis who achieved minimal disease activity: results from a Randomized, Double-Blind withdrawal study. *Arthritis Rheumatol.* **73**, 1663–1672 (2021). 33682378 10.1002/art.41716 PMC8457232

[8] Reich, A. et al. Real-world evidence for Ixekizumab in the treatment of psoriasis and psoriatic arthritis: literature review 2016–2021. *J. Dermatolog Treat.* **34**. (2023). 10.1080/09546634.2022.2160196 36629859

[9] Nash, P. et al. Ixekizumab for the treatment of patients with active psoriatic arthritis and an inadequate response to tumour necrosis factor inhibitors: results from the 24-week randomised, double-blind, placebo-controlled period of the SPIRIT-P2 phase 3 trial. *Lancet* **389**, 2317–2327 (2017). 28551073 10.1016/S0140-6736(17)31429-0

[10] Mease, P. J. et al. A head-to-head comparison of the efficacy and safety of Ixekizumab and adalimumab in biological-naïve patients with active psoriatic arthritis: 24-week results of a randomised, open-label, blinded-assessor trial. *Ann. Rheum. Dis.* **79**, 123–131 (2020). 31563894 10.1136/annrheumdis-2019-215386 PMC6937408

[11] Mease, P. J. et al. Ixekizumab, an interleukin-17A specific monoclonal antibody, for the treatment of biologic-naive patients with active psoriatic arthritis: results from the 24-week randomised, double-blind, placebo-controlled and active (adalimumab)-controlled period of the phase III trial SPIRIT-P1. *Ann. Rheum. Dis.* **76**, 79–87 (2017). 27553214 10.1136/annrheumdis-2016-209709 PMC5264219

[12] Bellis, E. et al. Retention rate of Ixekizumab in psoriatic arthritis: A Real-World study. *J. Pers. Med.* **14**, 716 (2024). 39063970 10.3390/jpm14070716 PMC11278385

[13] Braña, I. et al. Treatment retention and safety of Ixekizumab in psoriatic arthritis: A real life Single-Center experience. *J. Clin. Med.* ; **12**. (2023). 10.3390/jcm12020467 PMC9861177 36675395

[14] Chimenti, M. S. et al. Effectiveness of Ixekizumab over 24 months in different clinical scenarios in psoriatic arthritis: results from the gruppo Italiano studio early arthritis multicentric prospective registry. *Clin. Exp. Rheumatol.* **42** (11), 2221–2228 (2024). 10.55563/clinexprheumatol/udiit0 39051160

[15] Taylor, W. et al. Classification criteria for psoriatic arthritis: development of new criteria from a large international study. *Arthritis Rheum.* **54**, 2665–2673 (2006). 16871531 10.1002/art.21972

[16] Bruce, B. & Fries, J. F. The health assessment questionnaire (HAQ). *Clin. Exp. Rheumatol.* **23**, S14–S18 (2005). 16273780

[17] Schoels, M. M., Aletaha, D., Alasti, F. & Smolen, J. S. Disease activity in psoriatic arthritis (PsA): defining remission and treatment success using the DAPSA score. *Ann. Rheum. Dis.* **75**, 811–818 (2016). 26269398 10.1136/annrheumdis-2015-207507

[18] Michielsens, C. A. J. et al. Construct validity of bath ankylosing spondylitis disease activity index (BASDAI) and ankylosing spondylitis disease activity score (ASDAS) treatment target cut-offs in a BASDAI treat-to-target axial spondyloarthritis cohort: a cross-sectional study. *Scand. J. Rheumatol.* **53**, 180–187 (2024). 37339375 10.1080/03009742.2023.2213509

[19] Coates, L. C. et al. Original article: measurement properties of the minimal disease activity criteria for psoriatic arthritis. *RMD Open.* **5**, 1002 (2019). 10.1136/rmdopen-2019-001002 PMC6744081 31565243

[20] Coates, L. C. et al. Group for research and assessment of psoriasis and psoriatic arthritis (GRAPPA): updated treatment recommendations for psoriatic arthritis 2021. *Nat. Rev. Rheumatol.* **18**, 465–479 (2022). 35761070 10.1038/s41584-022-00798-0 PMC9244095

[21] Gossec, L. et al. EULAR recommendations for the management of psoriatic arthritis with Pharmacological therapies: 2023 update. *Ann. Rheum. Dis.* **83**, 706 (2024). 38499325 10.1136/ard-2024-225531 PMC11103320

[22] Yang, F. et al. Enthesitis in patients with psoriatic arthritis: A nationwide data from the Chinese registry of psoriatic arthritis (CREPAR). *Chin. Med. J. (Engl)*. **136**, 951 (2023). 37036901 10.1097/CM9.0000000000002646 PMC10278716

[23] Kaeley, G. S. et al. A hallmark of psoriatic arthritis. *Semin Arthritis Rheum.* **48**, 263–273 (2018). 29573849 10.1016/j.semarthrit.2018.02.002

[24] Poddubnyy, D., Jadon, D. R., Van den Bosch, F., Mease, P. J. & Gladman, D. D. Axial involvement in psoriatic arthritis: an update for rheumatologists. *Semin Arthritis Rheum.* **51**, 880–887 (2021). 34198146 10.1016/j.semarthrit.2021.06.006

[25] Chandran, V., Stecher, L., Farewell, V. & Gladman, D. D. Patterns of peripheral joint involvement in psoriatic arthritis-Symmetric, ray and/or row? *Semin Arthritis Rheum.* **48**, 430–435 (2018). 29724452 10.1016/j.semarthrit.2018.03.002 PMC6986918

[26] Kumthekar, A. & Ogdie, A. Obesity and psoriatic arthritis: A narrative review. *Rheumatol. Ther.* **7**, 447 (2020). 32495313 10.1007/s40744-020-00215-6 PMC7410935

[27] Caldarola, G. et al. Comparison of short- and long-term effectiveness of Ixekizumab and Secukinumab in real-world practice. *Expert Opin. Biol. Ther.* **21**, 279–286 (2021). 33170052 10.1080/14712598.2021.1849133

[28] Sherman, S. et al. Ixekizumab survival in heavily pretreated patients with psoriasis: A Two-year Single-centre retrospective study. *Acta Derm Venereol.* **100**, 1–5 (2020). 10.2340/00015555-3714 PMC9309699 33283248

[29] Rivera, R. et al. The effectiveness and safety of Ixekizumab in psoriasis patients under clinical practice conditions: A Spanish multicentre retrospective study. *Dermatol. Ther.* **33**, e14066 (2020). 32713119 10.1111/dth.14066

[30] Chiricozzi, A. et al. Ixekizumab effectiveness and safety in the treatment of Moderate-to-Severe plaque psoriasis: A Multicenter, retrospective observational study. *Am. J. Clin. Dermatol.* **21**, 441–447 (2020). 31786732 10.1007/s40257-019-00490-2

[31] Deza, G. et al. Initial results of Ixekizumab efficacy and safety in real-world plaque psoriasis patients: a multicentre retrospective study. *J. Eur. Acad. Dermatol. Venereol.* **33**, 553–559 (2019). 30317679 10.1111/jdv.15288

[32] Magdaleno-Tapial, J. et al. Efficacy and safety of Ixekizumab in a Real-Life practice: A retrospective bicentric study. *Actas Dermo-Sifiliográficas (English Edition)*. **110**, 585–589 (2019). 10.1016/j.ad.2019.02.006 31006480

[33] Tillett, W. et al. Changes in musculoskeletal disease activity and patient-reported outcomes in patients with psoriatic arthritis treated with ixekizumab: results from a real-world US cohort. *Front. Med. (Lausanne)*. **10**, 1184028 (2023). 37415769 10.3389/fmed.2023.1184028 PMC10322216

[34] Philip, A. K., Christopher, H. & Ritchlin, T. Psoriatic arthritis and burden of disease: patient perspectives from the Population-Based multinational assessment of psoriasis and psoriatic arthritis (MAPP) survey. *Rheumatol. Ther.* **3**. (2016). 10.1007/s40744-016-0029-z PMC4999579 27747516

[35] Walsh, J. A. et al. Impact of key manifestations of psoriatic arthritis on patient quality of life, functional status, and work productivity: findings from a real-world study in the united States and Europe. *Joint Bone Spine*. **90**, 105534 (2023). 36706947 10.1016/j.jbspin.2023.105534

[36] Bellinato, F., Gisondi, P., Fassio, A. & Girolomoni, G. Central pain sensitization in patients with chronic plaque psoriasis. *Dermatol. Ther. (Heidelb)*. **13** (5), 1149–1156 (2023). 36988902 10.1007/s13555-023-00917-z PMC10149419

[37] Jiang, X. et al. Interleukin-17 as a potential therapeutic target for chronic pain. **13**, 999407 (2022). 10.3389/fimmu.2022.999407 PMC9556763 36248896

[38] Deodhar, A. et al. The effect of Ixekizumab on axial manifestations in patients with psoriatic arthritis from two phase III clinical trials: SPIRIT-P1 and SPIRIT-P2. *Ther. Adv. Musculoskelet. Dis.* 15. (2023). 10.1177/1759720X231189005 PMC10462424 37645684

[39] Feld, J., Chandran, V., Haroon, N., Inman, R. & Gladman, D. Axial disease in psoriatic arthritis and ankylosing spondylitis: a critical comparison. *Nat. Rev. Rheumatol.* **14**, 363–371 (2018). 29752461 10.1038/s41584-018-0006-8

[40] Schmitt-Egenolf, M. et al. Drug persistence of biologic treatments in psoriasis: A Swedish National population study. *Dermatol. Ther. (Heidelb)*. **11**, 2107 (2021). 34661864 10.1007/s13555-021-00616-7 PMC8611161

[41] Lockshin, B. et al. Drug survival of ixekizumab, TNF inhibitors, and other IL-17 inhibitors in real-world patients with psoriasis: the Corrona psoriasis registry. *Dermatol. Ther.* **34**. (2021). 10.1111/dth.14808 PMC8047872 33491259

[42] Kemula, M., Morand, F., De Nascimento, J. & Sohrt, A. PBI58 persistence of treatment with biologics for patients with psoriasis: an analysis of a French prescription database. *Value Health*. **23**, S420 (2020).

[43] Leonardi, C. et al. Real-World biologic Adherence, Persistence, and monotherapy comparisons in US patients with psoriasis: results from IBM MarketScan^®^ databases. *Adv. Ther.* **39**, 3214–3224 (2022). 35570242 10.1007/s12325-022-02155-9 PMC9239953

[44] Blauvelt, A. et al. Comparison of Real-World treatment patterns among psoriasis patients treated with Ixekizumab or adalimumab. *Patient Prefer Adherence*. **14**, 517–527 (2020). 32210539 10.2147/PPA.S233993 PMC7074803

[45] Blauvelt, A. et al. Comparison of Real-World treatment patterns among Biologic-Experienced patients with psoriasis treated with Ixekizumab or Secukinumab over 18 months. *Dermatol. Ther. (Heidelb)*. **11**, 2133–2145 (2021). 34652590 10.1007/s13555-021-00627-4 PMC8611169

[46] Blauvelt, A. et al. A head-to-head comparison of Ixekizumab vs. guselkumab in patients with moderate-to-severe plaque psoriasis: 12-week efficacy, safety and speed of response from a randomized, double-blinded trial. *Br. J. Dermatol.* **182**, 1348–1358 (2020). 31887225 10.1111/bjd.18851 PMC7317420

[47] Tarannum, S. et al. Sex- and gender-related differences in psoriatic arthritis. *Nat. Rev. Rheumatol.* **18**, 513–526 (2022). 35927578 10.1038/s41584-022-00810-7

[48] Eder, L. et al. Responses to Ixekizumab in male and female patients with psoriatic arthritis: results from two Randomized, phase 3 clinical trials. *Rheumatol. Ther.* **9**, 919 (2022). 35397092 10.1007/s40744-022-00445-w PMC9127019

[49] Bartley, E. J. & Fillingim, R. B. Sex differences in pain: a brief review of clinical and experimental findings. *Br. J. Anaesth.* **111**, 52–60 (2013). 23794645 10.1093/bja/aet127 PMC3690315

[50] Cabo-Meseguer, A., Cerdá-Olmedo, G. & Trillo-Mata, J. L. Fibromyalgia: Prevalence, epidemiologic profiles and economic costs. *Med. Clin.* **149 2017** 441–448 . 10.1016/j.medcli.2017.06.008 28734619

[51] Pantano, I. et al. Secukinumab efficacy in patients with PsA is not dependent on patients’ body mass index. *Ann. Rheum. Dis.* **81**, e42–e42 (2022). 32169970 10.1136/annrheumdis-2020-217251

[52] Shahriari, M. et al. Disease response and patient-reported outcomes among initiators of Ixekizumab. *J. Dermatolog Treat.* **33**, 1538–1546 (2022). 33267635 10.1080/09546634.2020.1853023

[53] Reich, K. et al. The effect of bodyweight on the efficacy and safety of ixekizumab: results from an integrated database of three randomised, controlled phase 3 studies of patients with moderate-to-severe plaque psoriasis. *J. Eur. Acad. Dermatol. Venereol.* **31**, 1196–1207 (2017). 28370467 10.1111/jdv.14252

[54] Deodhar, A. et al. Long-term safety of Ixekizumab in adults with psoriasis, psoriatic arthritis, or axial spondyloarthritis: a post-hoc analysis of final safety data from 25 randomized clinical trials. *Arthritis Res. Ther.* **26**, 1–16 (2024). 38347650 10.1186/s13075-023-03257-7 PMC10860236

[55] Targan, S. R. et al. A Randomized, Double-Blind, Placebo-Controlled phase 2 study of brodalumab in patients with Moderate-to-Severe crohn’s disease. *Am. J. Gastroenterol.* **111**, 1599–1607 (2016). 27481309 10.1038/ajg.2016.298

[56] Hueber, W. et al. Secukinumab, a human anti-IL-17A monoclonal antibody, for moderate to severe crohn’s disease: unexpected results of a randomised, double-blind placebo-controlled trial. *Gut* **61**, 1693 (2012). 22595313 10.1136/gutjnl-2011-301668 PMC4902107

[57] Smith, M. K. et al. Crohn’s-like disease in a patient exposed to anti-Interleukin-17 Blockade (Ixekizumab) for the treatment of chronic plaque psoriasis: a case report. *BMC Gastroenterol.* **19**, 162 (2019). 31488067 10.1186/s12876-019-1067-0 PMC6727530

